# Supplementary material for: A natural experiment in Kenya reveals durable immunosuppressive effects of early childhood malaria: a longitudinal cohort study
Source: eLife. 2026 Jul 14;14:RP107820. doi: 10.7554/eLife.107820 (PMC13368175; doi:10.7554/eLife.107820)
Supplement: Supplementary file 1. [file elife-107820-supp1.docx]

| **Pathogen** | **Strain/subtype** | **Antigen** |
| --- | --- | --- |
| Measles virus | Edmonston | Whole virus antigen |
| Coxsackie B virus | B1 | VP1 |
| Cytomegalovirus | TB40E | PP150 |
| Epstein-Barr virus | Type 1 | Nuclear antigen 1 |
| Herpes simplex virus | Type 1 | Extract |
| H1N1 Influenza A | A/California/07/2009 | Haemagluttinin |
| Plasmodium falciparum | 3D7 | AMA-1 |
| Rubella virus | HPV-77 | Whole virus antigen |
