# Supplementary material for: A natural experiment in Kenya reveals durable immunosuppressive effects of early childhood malaria: a longitudinal cohort study
Source: eLife. 2026 Jul 14;14:RP107820. doi: 10.7554/eLife.107820 (PMC13368175; doi:10.7554/eLife.107820)
Supplement: Reporting standard 1. [file elife-107820-repstand1.pdf]

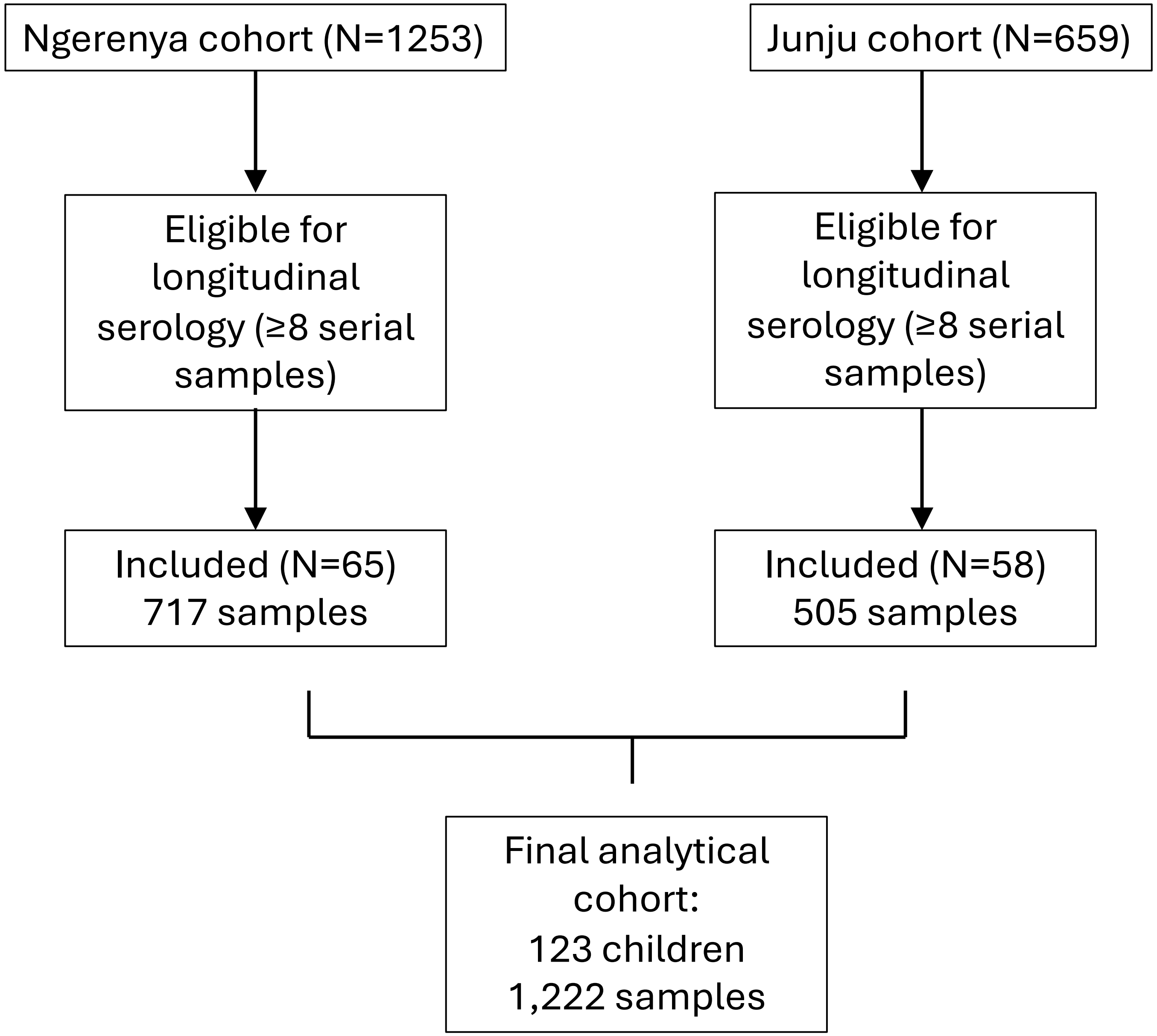
Ngerenya cohort (N=1253)
Junju cohort (N=659)
Eligible for longitudinal serology (≥8 serial samples)
Eligible for longitudinal serology (≥8 serial samples)
Included (N=65)
717 samples
Included (N=58)
505 samples
Final analytical cohort:
123 children
1,222 samples
