# Supplementary material for: A natural experiment in Kenya reveals durable immunosuppressive effects of early childhood malaria: a longitudinal cohort study
Source: eLife. 2026 Jul 14;14:RP107820. doi: 10.7554/eLife.107820 (PMC13368175; doi:10.7554/eLife.107820)
Supplement: Peer review history

# Editor report: eLife Assessment

**DOI:** 10.7554/eLife.107820.3.sa0

**Authors:** Marcelo U Ferreira

**Affiliations:** University of São Paulo Brazil

**Keywords:** Important, Solid

This **important** study provides **solid** evidence that early childhood malaria exposure affects the development of antibody responses to unrelated pathogens and vaccine-derived antigens in Kenyan children. The findings are of major public health importance and limitations of the observational study design are properly acknowledged.

# Referee report: Reviewer #2 (Public review)

**DOI:** 10.7554/eLife.107820.3.sa1

Summary:

The authors investigated whether early-life malaria exposure has long-term effects on immune responses to unrelated antigens. They leveraged a natural experiment in coastal Kenya where two adjacent communities (Junju and Ngerenya) experienced divergent malaria transmission patterns after 2004. Using 15 years of longitudinal data from 123 children with weekly malaria surveillance and annual serological sampling, they measured antibody responses to multiple pathogens using a protein microarray technology and ELISA.

Strengths:

(1) Extensive longitudinal data collection with weekly malaria surveillance, enabling precise exposure classification.

(2) Use of a natural experiment design that allows for causal inference about malaria's immunological effects.

(3) Broad panel of antigens tested, demonstrating generalized rather than antigen-specific effects.

(4) Within-cohort analysis in Ngerenya controls for geographic and environmental factors.

(5) Validation of key findings using both serologic microarray and ELISA.

(6) Important public health implications for vaccine strategies in malaria-endemic regions.

Weaknesses:

(1) Due to its nature, the study lacks the ability to determine the direction of the associations found between malaria exposure and other IgG levels to unrelated pathogens.

(2) No evaluation of the clinical Implications of the reduced IgG levels observed in the area with high malaria exposure.

Assessment of Claims:

The data appear to support the authors' primary claims. The strength of the evidence is limited by the observational nature of the study and the results should be interpreted in that light. Together with the currently available evidence of *P. falciparum*'s impact on the host's immune function, this natural experiment design provides further evidence for a relationship between early malaria exposure and reduced antibody responses to other pathogens and vaccine-derived antigens. The within-Ngerenya analysis controls for geographic factors and thus enhances the quality of the evidence; there is limited physical, nutritional, and socio-economic information on factors that may have driven the observed changes.

Impact and Utility:

This work has fundamental implications for understanding vaccine effectiveness in malaria-endemic regions and may contribute to inform vaccination strategies. The findings, if confirmed, would suggest that children in areas of high malaria transmission may require modified immunization approaches. The dataset provides a valuable resource for future studies of malaria's immunological legacy.

Context:

This study builds on prior work showing acute immunosuppressive effects of malaria but uniquely attempts to demonstrate the durability of these effects years after exposure. The natural experiment design addresses limitations of previous observational studies by providing a more controlled comparison.

# Author comment: Author response

**DOI:** 10.7554/eLife.107820.3.sa2

**Authors:** Mercy S Safari, Timothy Makori, Elijah T Gicheru, Maureen W Mburu, Omar K Nyawa, Faiz M Shee, James Nyagwange, E Wangeci Kagucia, Francis Maina Ndungu, Timothy Chege Kuria, James O Tuju, Charles J Sande

**Affiliations:** Kenya Medical Research Institute Kilifi Kenya; Kenya Medical Research Institute (KEMRI)-Wellcome Trust Research Programme Kilifi Kenya; Kenya Medical Research Institute (KEMRI)-Wellcome Trust Research Programme Kilifi Kenya; Kenya Medical Research Institute-Wellcome Trust Research Programme Kilifi Kenya; Kenya Medical Research Institute-Wellcome Trust Research Programme Kilifi Kenya; Pwani University Kilifi Kenya; Kenya Medical Research Institute-Wellcome Trust Research Programme Kilifi Kenya; KEMRI-Wellcome Trust Research Programme Kilifi Kenya; Kenya Medical Research Institute Kilifi Kenya; Columbia University Irving Medical Center, New York Kilifi Kenya; KEMRI-Wellcome Trust Research Programme Kilifi Kenya; KEMRI-Wellcome Research Programme; Kilifi Kenya

The following is the authors’ response to the original reviews.

**eLife Assessment**

This important study sought to investigate the role that early childhood malaria exposure plays in the development of antibody responses to unrelated pathogens and vaccine-derived antigens in Kenyan children. In this natural experiment, the authors compare antibody levels among children who have been exposed to different levels of malaria transmission by using protein microarray technology. Although the findings are of importance, the evidence remains incomplete, and the analysis would benefit from a more in-depth evaluation of potential confounders. With the appropriate analysis, the findings will be of great interest for global health, immunology, and vaccine development.

We thank the editors for highlighting the need for a more comprehensive evaluation of potential confounding. We agree that this is a critical aspect of the study and have now undertaken additional analyses to address this directly.

The original longitudinal cohort was designed to investigate the acquisition of naturally acquired immunity to malaria and did not include systematic collection of anthropometric/nutritional, environmental or socioeconomic data, precluding direct adjustment for these factors within the primary dataset. However, to assess whether there were population-level differences in these factors, we leveraged contemporaneous hospital-based surveillance data from the same geographic regions, which includes measurements of anthropometry and nutritional status (muac, weight-for-age, and height-for-age) and detailed infection diagnostics.

Using this independent dataset, we fitted mixed-effects regression models adjusting for age, calendar year, and concurrent infections (RSV, parainfluenza, influenza A, human metapneumovirus, OC43). For all three anthropometric indices, we found no evidence of systematic differences between children from Junju and Ngerenya. Adjusted differences were small and centred around zero (muac: −0.12, 95% CI −0.38 to 0.15, weight-for-age: −0.05, −0.28 to 0.19, height-for-age: 0.08, −0.17 to 0.33), with no consistent directional effect.

As the longitudinal cohort was randomly selected from these underlying populations, these findings suggest that the groups were broadly comparable with respect to nutritional status and there were no differences in their exposure to the infections that were included in the analysis. We have incorporated these analyses into the revised manuscript, added a new figure focussed on this analysis -fig. 6, updated the statistical analysis and discussion sections, and believe they substantially strengthen the evidence by addressing a key source of potential confounding.

**Public Reviews:**

**Reviewer #1 (Public review):**

Summary:

The study shows that childhood malaria can weaken the antibody response to other vaccines and infections. This suggests that early exposure to *P. falciparum* may have a long-lasting effect on immunity, with implications for vaccine efficacy in endemic areas.

Strengths:

This study stands out for its longitudinal design, the use of robust immunological techniques, and the comparison between areas with different levels of malaria exposure. Its findings reveal that early malaria can weaken the response to childhood vaccines, with important implications for public health in endemic regions.

We thank the reviewer for this comment

Weaknesses:

One of the study's main limitations is the lack of functional data confirming the clinical impact of the low antibody levels. Furthermore, although multiple immune responses were measured, other important components, such as cellular immunity, were not assessed. Furthermore, the results may not be generalizable to other regions.

We thank the reviewer for this important comment and agree that the absence of functional immunological assays is a limitation of the current study. Our analysis was designed to determine whether early-life malaria exposure is associated with durable alterations in antibody responses to unrelated pathogens and vaccine antigens, rather than to establish the downstream functional consequences of these differences. As such, the study is able to demonstrate a broad and persistent attenuation of humoral responses but cannot directly determine whether the lower antibody levels observed translate into reduced neutralising capacity or diminished protection at the individual level.

We have revised the manuscript to make this distinction more explicit. In the revised discussion, we now state that although reduced antibody titres to vaccine-preventable pathogens may have implications for long-term protection, the clinical significance of these differences remains to be established in future studies incorporating functional assays and clinical outcome data.

**Reviewer #2 (Public review):**

Summary:

The authors investigated whether early-life malaria exposure has long-term effects on immune responses to unrelated antigens. They leveraged a natural experiment in coastal Kenya where two adjacent communities (Junju and Ngerenya) experienced divergent malaria transmission patterns after 2004. Using 15 years of longitudinal data from 123 children with weekly malaria surveillance and annual serological sampling, they measured antibody responses to multiple pathogens using a protein microarray technology and ELISA.

Strengths:

(1) Extensive longitudinal data collection with weekly malaria surveillance, enabling precise exposure classification.

(2) Use of a natural experiment design that allows for causal inference about malaria's immunological effects.

(3) Broad panel of antigens tested, demonstrating generalized rather than antigen-specific effects.

(4) Within-cohort analysis in Ngerenya controls for geographic and environmental factors.

(5) Validation of key findings using both serologic microarray and ELISA.

(6) Important public health implications for vaccine strategies in malaria-endemic regions.

We thank the reviewer for these comments

Weaknesses:

(1) Lack of participants' characteristics (socio-economic, nutritional, physical).

We thank the reviewer for this important comment. We have now included a detailed summary of participant characteristics in Table 1to provide context for the study population. This includes key demographic and longitudinal variables stratified by cohort (Junju and Ngerenya), including sex distribution, age at study entry and exit, duration of follow-up, number of visits per participant, and total serum samples analysed. Detailed data on socio-economic status, nutritional status, and other environmental or physical characteristics were not consistently available across all participants and time points, and therefore could not be included. This has now been explicitly stated as a limitation in the discussion.

(2) Somewhat limited sample size (longitudinal analysis of 123 children total), with further subdivision reducing statistical power for some analyses.

We thank the reviewer for this important observation. The study is based on an intensively followed cohort with weekly malaria surveillance and repeated serological measurements throughout childhood, allowing detailed characterisation of early-life exposure and subsequent immune trajectories. This depth of longitudinal sampling provides resolution that is not achievable in larger cross-sectional studies. We acknowledge that subdivision of the cohort reduces statistical power for some analyses. Nevertheless, the key findings were consistent in several independent comparisons, including a reduction in antibody levels for broad panel of antigens in the malaria endemic setting, within-cohort analyses in Ngerenya that replicated this observation, and the confirmation of results generated on the protein microarray on the ELISA platform. The consistency of these findings across analytical approaches and measurement platforms reduces the likelihood that the observed effects are driven by small-sample variability. We have clarified this point in the revised discussion to emphasise that the strength of the study lies in the depth and longitudinal resolution of the data rather than the absolute sample size.

(3) Potential confounding by unmeasured socioeconomic, nutritional, or environmental factors between communities.

We thank the reviewer for this important point and agree that residual confounding between communities must be considered. As outlined in reponse to the editorial assesment, we have undertaken additional analyses using contemporaneous population-level data from the same regions and found no evidence of systematic differences in anthropometric indices between children from Junju and Ngerenya after accounting for age, calendar year, and concurrent infections, with effect estimates small and crossing zer. In addition, the within-Ngerenya analysis provides an internal comparison within a shared geographic and environmental setting, reducing the likelihood that unmeasured socioeconomic or environmental differences between communities account for the observed associations. The new analyses suggest that major population-level differences in nutritional status or infection burden are unlikely to explain the observed patterns. We have clarified this point in the revised discussion and explicitly acknowledge the possibility of residual confounding.

(4) Lack of ability to determine the direction of the associations found between malaria exposure and other IgG levels to unrelated pathogens.

We agree that, as an observational study, our analysis cannot definitively establish the direction of the association between malaria exposure and antibody responses to unrelated antigens. However, several features of the study design strengthen the inference that early-life malaria exposure contributes to the observed differences. First, malaria exposure was characterised prospectively through intensive weekly surveillance, allowing precise temporal definition of exposure in early childhood. Second, within the Ngerenya cohort, where children were exposed to different levels of malaria due to a rapid decline in transmission, those with even limited early-life exposure exhibited lower antibody responses at 10 years of age than malaria-naïve peers, despite residing in the same geographic and environmental context. In addition, we now show that these differences are not confined to a single timepoint but are evident across the full longitudinal follow-up after adjustment for age and repeated measurements. While we cannot exclude the possibility of residual confounding or bidirectional relationships, the convergence of evidence from the natural experiment design, within-cohort contrasts, and age-adjusted longitudinal analyses supports early-life malaria exposure as a key contributor to long-term differences in antibody responses. We have clarified this in the discussion.

(5) Despite good longitudinal data, the main analysis was conducted as a cross-sectional analysis at age 10 for many comparisons, which limits the understanding of temporal dynamics.

We thank the reviewer for highlighting this point. While age 10 was initially used as a standardised reference point for cross-sectional comparisons, the underlying dataset is longitudinal, with repeated antibody measurements across childhood. To address this more directly, we have now complemented these analyses with antigen-specific mixed-effects regression models incorporating all available longitudinal data, with adjustment for age and a random intercept for repeated measurements within individuals. These models demonstrate that the differences between cohorts are not confined to the age-10 cross-section but are evident in an age-adjusted longitudinal framework for multiple antigens. We have retained the age-10 comparisons for reference, but the primary inference is now based on the longitudinal mixed-effects analyses. These changes are reflected in the revised results and statistical analysis sections. We thank the reviewer for this astute point, which we think has substantially improved the manuscript.

(6) Statistical analysis is limited to univariable comparisons without consideration for confounders or adjusting for multiple comparisons.

We agree that the original analyses relied primarily on univariable comparisons. In the revised manuscript, we have extended the analytical framework to include mixed-effects regression models that account for age effects and repeated measurements within individuals. These models estimate the average age-adjusted difference in antibody responses between cohorts across the full follow-up period. We have also applied false discovery rate (FDR) correction to account for multiple antigen testing. For multiple antigens, the direction and magnitude of cohort differences remain consistent under this approach, strengthening the robustness of the findings beyond the original univariable comparisons. These analyses have been incorporated into the revised results and statistical analysis sections.

(7) No mechanistic understanding of how early malaria exposure creates lasting immunosuppression.

We agree that this study does not directly resolve the mechanistic basis underlying the observed long-term differences in antibody responses. The primary aim of this work was to identify and characterise durable alterations in humoral immune profiles associated with early-life malaria exposure, rather than to define the cellular or molecular pathways involved. However, our findings are consistent with a growing body of experimental and clinical literature suggesting that malaria infection can induce sustained perturbations in B cell and T cell compartments, including the expansion of atypical memory B cells, altered germinal centre responses, and increased regulatory immune activity. These mechanisms have been proposed to impair the generation and maintenance of effective humoral immunity. In the revised discussion, we have clarified that the mechanistic basis of this phenomenon remains to be fully defined and have expanded the discussion of plausible pathways in light of existing literature. We now explicitly position our findings as providing population-level evidence of a durable immunological phenotype that warrants further mechanistic investigation.

(8) No understanding of the clinical Implications of the reduced IgG levels observed in the area with high malaria exposure.

We agree that this study does not directly establish the clinical consequences of the reduced antibody levels observed in malaria-exposed children. The primary objective of this study was to characterise long-term differences in humoral immune profiles associated with early-life malaria exposure, rather than to assess downstream clinical outcomes or functional antibody activity. We have clarified this limitation in the revised discussion. Nevertheless, the breadth and consistency of the observed differences for multiple vaccine-preventable and infectious antigens raise the possibility that early-life malaria exposure may have implications for long-term immune protection. We now emphasise in the revised discussion that future studies incorporating functional assays and clinical outcome data will be required to determine whether these serological differences translate into altered susceptibility to infection or reduced vaccine effectiveness.

Assessment of Claims:

The data appear to support the authors' primary claims, but the strength of the evidence is limited, and the results should be interpreted with caution. Together with the currently available evidence of *P. falciparum*'s impact on the host's immune function, this natural experiment design provides further evidence for a relationship between early malaria exposure and reduced antibody responses. The within-Ngerenya analysis controls for geographic factors and thus enhances the quality of the evidence, however, it still fails to account for the physical, nutritional, and socio-economic factors that may have driven the observed changes. Additionally, the mechanism underlying this effect remains unclear, and the clinical significance of reduced antibody levels is not established.

We thank the reviewer for this assessment and for recognising the strengths of the natural experiment design and within-cohort analyses. We agree that, as an observational study, our findings should be interpreted appropriately. In the revised manuscript, we have undertaken additional analyses and clarifications to strengthen the evidential basis of our conclusions and to address the points raised. To address potential confounding by nutritional and related factors, we analysed contemporaneous hospital-based surveillance data from the same geographic populations since nutritional and socioeconomic variables were not consistently collected during the course of longitudinal follow up. For three independent anthropometric indices of nutrition status (muac, weight-for-age, and height-for-age), we found no evidence of systematic differences between children from Junju and Ngerenya after adjustment for age, calendar year, and concurrent infections. As the longitudinal cohort subjects were randomly drawn from these populations, these findings suggest that the two groups were broadly comparable with respect to early-life growth and nutritional status.

We agree that the mechanistic basis of the observed differences is not resolved in this observational study. We have clarified this point in the revised discussion and expanded our consideration of plausible biological pathways based on existing literature, including perturbations in B cell and T cell compartments. Similarly, we now explicitly state that the clinical implications of reduced antibody levels remain to be determined and will require studies incorporating functional assays and clinical outcomes. We believe these revisions strengthen the manuscript by providing a more comprehensive interpretation of the data.

Impact and Utility:

This work has fundamental implications for understanding vaccine effectiveness in malaria-endemic regions and may contribute to informing vaccination strategies. The findings, if strengthened, would suggest that children in areas of high malaria transmission may require modified immunization approaches. The dataset provides a valuable resource for future studies of malaria's immunological legacy.

We thank the reviewer for this comment

Context:

This study builds on prior work showing acute immunosuppressive effects of malaria but uniquely attempts to demonstrate the durability of these effects years after exposure. The natural experiment design addresses limitations of previous observational studies by providing a more controlled comparison.

We thank the reviewer for this comment

**Recommendations for the authors:**

**Reviewing Editor Comments:**

We suggest that further analyses of potential confounders such as anthropometric indices, socioeconomic status, and comorbidities would render the evidence more robust.

We thank the Reviewing Editor for this important suggestion. We agree that careful consideration of potential confounding factors is critical to the interpretation of these findings, and have undertaken additional analyses to address this.

Because anthropometric and related socioeconomic measurements were not collected systematically within the original longitudinal malaria cohort, we assessed potential population-level differences using hospital-based surveillance data from the same geographic regions. This dataset includes measurements of anthropometry (mid-upper arm circumference, weight-for-age, and height-for-age) as well as detailed infection diagnostics in childhood. Using these data, we fitted regression models adjusting for age, calendar year, and concurrent, clinically diagnosed infections. For all three anthropometric indices, we found no evidence of systematic differences between children from Junju and Ngerenya, with effect estimates small and crossing zero (fig. 6). As the longitudinal cohorts were randomly selected from these populations, these findings suggest that the groups were broadly comparable with respect to nutritional status and infection exposure. With respect to socioeconomic status and comorbidities, detailed individual-level data were not available within the longitudinal cohort. However, the within-Ngerenya analysis, where children with differing early-life malaria exposure were compared within the same geographic and environmental setting, provides a complementary control for these factors. We have incorporated these additional analyses and clarifications into the revised manuscript statistical analysis, discussion lines and believe they strengthen the robustness of the findings by addressing key sources of potential confounding.

**Reviewer #1 (Recommendations for the authors):**

The manuscript is well written, with clear and informative figures that effectively support the findings.

We thank the reviewer for this comment

Suggestions:

(1) Although the study well controlled for malaria exposure, other environmental or infectious factors that influence immunity could be considered:

Nutritional status in childhood (malnutrition impacts immune response), co-infections (helminths, respiratory viruses), socioeconomic differences, or differences in access to health services, even minimal, between Junju and Ngerenya.

We thank the reviewer for highlighting the potential influence of environmental, infectious, and socioeconomic factors on immune responses. We agree that these are important considerations in the interpretation of observational data. To address nutritional status and concurrent infectious exposures, we analysed contemporaneous hospital-based surveillance data from the same geographic populations. This dataset includes measurements of anthropometric indices (mid-upper arm circumference, weight-for-age, and height-for-age) alongside detailed clinical diagnostics for common childhood infections. Using regression models adjusting for age, calendar year, and concurrent infections, we found no evidence of systematic differences in anthropometric profiles between children from Junju and Ngerenya (fig. 6). These findings suggest that the populations from which the longitudinal cohorts were randomly selected were comparable with regard to early-life growth and nutritional status. We agree that we cannot fully exclude the influence of unmeasured factors such as helminth infections, socioeconomic variation, or subtle differences in healthcare access. However, the within-Ngerenya analysis, where children with differing early-life malaria exposure were compared within the same geographic, environmental, and healthcare setting, provides an internal control for many of these factors. The persistence of similar patterns within this setting supports malaria exposure as a key contributor of the observed differences. We have clarified these considerations in the revised discussion and believe that, the additional analyses and within-cohort comparisons strengthen the robustness of our conclusions while acknowledging the limitations inherent to observational studies.

(2) Measurement of other immunological markers:

In addition to IgG, include: B cell subpopulations (naive, memory, atypical), cytokine levels (IL-10, IFN-γ) to characterize the immunological microenvironment.

You could include these recommendations in the text for future studies.

We thank the reviewer for this thoughtful suggestion. We agree that detailed immunophenotyping, including characterisation of B cell subpopulations and cytokine profiles, would provide important insight into the mechanisms underlying the observed differences in antibody responses. In the revised manuscript, we have expanded the discussion to highlight these important avenues for future work, including the potential role of altered B cell subsets (and regulatory or inflammatory cytokine environments in shaping long-term humoral responses).

**Reviewer #2 (Recommendations for the authors):**

The manuscript is well-written.

We thank the reviewer for this comment

(1) Methodological Clarifications:

Do the authors have any information regarding the characteristics of these children that could be of use in understanding their immune responses better? (e.g., weight, height, BMI, socioeconomic status, HB level, access to health care, etc.).

We thank the reviewer for highlighting the importance of participant characteristics in interpreting immune responses. Anthropometric and related clinical measures were not collected systematically within the original longitudinal malaria cohort, as the study was designed to investigate the acquisition of naturally acquired immunity to malaria.

To address this, we analysed contemporaneous hospital-based surveillance data from the same geographic populations, which include measurements of anthropometric indices (mid-upper arm circumference, weight-for-age, and height-for-age) alongside detailed infection diagnostics. Using regression models adjusting for age, calendar year, and concurrent infections, we found no evidence of systematic differences in anthropometric profiles between children from Junju and Ngerenya (fig. 6) Detailed individual-level data on socioeconomic status, haemoglobin levels, and healthcare access were not available within the longitudinal cohort impeding direct adjustment in the longitudinal cohorts. However, the within-Ngerenya analysis, where children with differing early-life malaria exposure were compared within the same geographic and healthcare setting, provides an internal control for many of these factors. These considerations are now clarified in the revised discussion.

Could the authors provide more detailed statistical analysis, including power calculations and multiple comparison corrections?

In the revised manuscript, we have extended the statistical analysis and now include antigen-specific mixed-effects regression models incorporating all available longitudinal measurements, which is comprehensively described in the statistical analysis section. We have also applied false discovery rate (FDR) correction to account for multiple testing across antigens, and report both unadjusted and FDR-adjusted significance in the revised results. With respect to power, the sample size was determined by the number of children meeting inclusion criteria within the long-term surveillance cohorts in terms of availability of a sufficient number of longitudinal samples. We have clarified this in the revised manuscript.

Clarify the criteria for selecting the 123-child subset from the larger surveillance cohorts.

We thank the reviewer for this comment. The 123 children included in this analysis were selected from the larger surveillance cohorts based on the availability of sufficiently dense longitudinal serum sampling as described above. Specifically, children were required to have at least eight longitudinal samples available in the archive, enabling robust assessment of within-individual antibody trends over time. This criterion was applied to ensure adequate temporal resolution to examine the long-term stability of malaria-associated effects on antibody responses. Children with fewer available samples were therefore excluded, as limited sampling would not allow reliable characterisation of longitudinal patterns. We have clarified these inclusion criteria in the revised manuscript.

(2) Additional Analyses and Data Presentation:

The authors could consider dose-response analyses relating malaria episode frequency/timing to degree of immunosuppression or even AMA-1 IgG levels and degree of immunosuppression. How do they associate over time?

We thank the reviewer for this suggestion. To address this, we examined the relationship between malaria exposure (using cumulative febrile malaria episode count derived from longitudinal surveillance data) and the magnitude of heterologous antibody responses. In mixed-effects models adjusting for age and repeated antibody measurements, higher malaria episode burden was associated with lower antibody responses against multiple antigens (fig 7).

Analyze whether the effects vary by specific age at malaria exposure.

We agree that age at exposure is an important consideration. We have now assessed how the relationship between malaria burden and antibody responses varies with age by including age as a non-linear term and modelling interactions between malaria exposure and age as described above. These analyses did not suggest substantial heterogeneity in the association over age, and therefore we have retained the simpler presentation for clarity.

Provide correlation analyses between different antibody responses to assess whether suppression is generalized.

We have addressed this by modelling responses jointly across a panel of heterologous antigens and by examining antigen-specific associations. The direction of effect was consistent for the majority of antigens, with no evidence of opposing trends, supporting a broad rather than antigen-specific effect.

The authors could consider moving Figures 2a and b to the supplementary material.

We thank the reviewer for this suggestion. We carefully considered whether panels 2a and 2b could be moved to the supplementary material. However, we have retained them in the main text because they provide a simple, intuitive illustration of how AMA1 antibody responses track with malaria exposure at the individual level, complementing the population-level analysis shown in fig. 2c. We feel that this helps establish the biological validity of the microarray platform in a way that is immediately interpretable to the reader, and therefore supports the interpretation of subsequent analyses.

The authors could consider replacing Figures 3a and b with IgG levels from ALL vaccinated children and ALL non-vaccinated children.

We thank the reviewer for this suggestion. We would like to retain these figures for the same reasons that have been articulated above for figures 2a and b.

(3) Discussion Enhancements:

The authors should consider expanding the discussion to address the limitations of the data more thoroughly, particularly regarding the potential differences between cohorts that could have contributed to the results.

We have expanded the discussion to more explicitly address potential differences between cohorts that could contribute to the observed findings, including nutritional, socioeconomic, and environmental factors.

The discussion needs to acknowledge the lack of directionality for the associations observed. As stated above, although I agree in general terms with the observations that the authors have made, it is not possible to distinguish between a suppressive effect of malaria on immune responses to infection-derived pathogens or a protective effect of malaria that leads to less exposure to infection-derived pathogens (and consequently lower IgG levels). The mechanisms behind these could include things like different health-seeking behaviors or social interactions from kids who have malaria versus those who don't, for example.

We agree that, as an observational study, we cannot definitively establish the direction of the association between malaria exposure and antibody responses to unrelated antigens. We have now clarified this limitation explicitly in the discussion. We acknowledge the alternative interpretations raised by the reviewer, including the possibility that differences in exposure to other pathogens, potentially driven by behavioural, environmental or healthcare-related factors, could contribute to the observed patterns. At the same time, we note that the natural experiment design, prospective malaria exposure classification, and within-Ngerenya comparisons support early-life malaria exposure as a key contributing factor. We have revised the discussion to reflect this balance.

Extend the discussion of potential biological mechanisms underlying durable immunosuppression.

We thank the reviewer for this suggestion. We have expanded the discussion to more fully consider potential biological mechanisms that could underlie the observed long-term differences in antibody responses. Specifically, we now discuss evidence from prior studies indicating that malaria infection can induce sustained alterations in B cell and T cell compartments, including expansion of atypical memory B cells, disruption of germinal centre responses, and increased regulatory immune activity. We position our findings as providing population-level evidence of a durable immunological phenotype, while noting that targeted mechanistic studies will be required to define the underlying pathways.

Extend the discussion around the clinical implications of the observed antibody level differences.

In the revised discussion, we highlight that studies incorporating functional assays and clinical outcome data will be required to determine whether these serological differences translate into altered susceptibility to infection or reduced vaccine effectiveness.

(4) Technical Issues:

Could the authors please:

(1) Clarify microarray data processing and quality control procedures.

We thank the reviewer for this request. We have expanded the methods section to provide additional detail on microarray data processing and quality control procedures.

(2) Provide information on inter-assay variability and batch effects.

We have expanded the methods section to clarify how these were evaluated and addressed. Inter-assay variability was monitored using pooled adult serum included on every slide as a consistent positive control. This allowed us to assess slide-to-slide consistency in signal detection across the full antigen panel. In addition, fluorophore-conjugated IgG and IgA controls were printed directly onto each miniarray to confirm scanner performance independently of antigen–antibody interactions. At the sample level, each specimen was assayed on two independent miniarrays per slide, generating four spatially separated replicate measurements per antigen. Technical variability was quantified using the coefficient of variation (CV), and measurements with CV >20% were excluded from downstream analyses.

(3) Include details on how missing data were handled in longitudinal analyses.

We thank the reviewer for highlighting this point. We have added clarification in the statistical analysis section describing how missing data were handled. Specifically, mixed-effects models were used, which accommodate unbalanced longitudinal data without requiring imputation, allowing all available observations to contribute to the analysis.

(4) Include details of the parameters of the LOWESS analysis shown in Figure 1.

We have expanded the figure 1 legend to include the parameters used for the loess smoothing shown, including the smoothing span.

(5) Include details of the samples used for Figure 3d (Negative and Pooled Adult Serum).

We have clarified in the methods the nature and purpose of the samples used in Figure 3d. The negative control consisted of phosphate-buffered saline applied to a full miniarray in place of serum, allowing assessment of background and non-specific signal in the absence of antibody binding. The pooled adult serum comprised a composite of sera from multiple healthy adults from the same setting and was included as a positive reference sample, expected to contain a broad repertoire of antigen-specific antibodies. These controls were included on each slide to enable interpretation of assay performance, with the negative control defining baseline signal and the pooled adult serum providing a consistent reference for antigen recognition across the microarray.
